# Computational prediction of molecular pathogen-host interactions based on dual transcriptome data

**DOI:** 10.3389/fmicb.2015.00065

**Published:** 2015-02-06

**Authors:** Sylvie Schulze, Sebastian G. Henkel, Dominik Driesch, Reinhard Guthke, Jörg Linde

**Affiliations:** ^1^Department of Systems Biology and Bioinformatics, Leibniz-Institute for Natural Product Research and Infection Biology – Hans-Knoell-InstituteJena, Germany; ^2^BioControl Jena GmbHJena, Germany

**Keywords:** network inference, NetGenerator, transcriptomics, dual RNA-Seq, microarrays, gene regulatory networks, inter-species interactions

## Abstract

Inference of inter-species gene regulatory networks based on gene expression data is an important computational method to predict pathogen-host interactions (PHIs). Both the experimental setup and the nature of PHIs exhibit certain characteristics. First, besides an environmental change, the battle between pathogen and host leads to a constantly changing environment and thus complex gene expression patterns. Second, there might be a delay until one of the organisms reacts. Third, toward later time points only one organism may survive leading to missing gene expression data of the other organism. Here, we account for PHI characteristics by extending NetGenerator, a network inference tool that predicts gene regulatory networks from gene expression time series data. We tested multiple modeling scenarios regarding the stimuli functions of the interaction network based on a benchmark example. We show that modeling perturbation of a PHI network by multiple stimuli better represents the underlying biological phenomena. Furthermore, we utilized the benchmark example to test the influence of missing data points on the inference performance. Our results suggest that PHI network inference with missing data is possible, but we recommend to provide complete time series data. Finally, we extended the NetGenerator tool to incorporate gene- and time point specific variances, because complex PHIs may lead to high variance in expression data. Sample variances are directly considered in the objective function of NetGenerator and indirectly by testing the robustness of interactions based on variance dependent disturbance of gene expression values. We evaluated the method of variance incorporation on dual RNA sequencing (RNA-Seq) data of *Mus musculus* dendritic cells incubated with *Candida albicans* and proofed our method by predicting previously verified PHIs as robust interactions.

## 1. Introduction

Organisms need to constantly adapt to environmental changes. On a molecular level, this is mediated by complex signaling cascades, which transmit the signal to cell nuclei. Transcription factors bind to their target genes, which consequently leads to a change in gene expression. This way, biological systems adapt to new environmental conditions.

In most cases underlying networks are unknown. This is especially interesting for interacting organisms, such as pathogens and host. Both the experimental setup and the nature of PHIs exhibit certain characteristics: (i) pathogen and host are in a battle leading to constantly changing conditions, (ii) a change in gene expression is triggered by new environmental conditions and the response of one organism might initiate faster or persist longer than the response of the other organism and (iii) two different organisms interact and eventually one survives which can lead to missing data time points.

The immune system of the host is permanently active to recognize and eliminate infectious microorganisms. As a first line of defense, components of the innate immune system such as the complement system, immune cells, and antimicrobial peptides recognize pathogen-associated molecular patterns (PAMPs). In contrast, pathogens developed many strategies to evade these mechanisms. They can shield microbe-associated cell surface proteins, mimic host surfaces or secrete proteases degrading host immune proteins (Zipfel et al., 2011). Nevertheless, the interaction with host cells is also important for pathogens, e.g., to acquire nutrients and to replicate (Casadevall and Pirofski, 2000).

The transcriptome of pathogen and host can be measured by physical separation of pathogen and host cells before RNA extraction. This enables RNA extraction from pathogen and host at different time points. For example, Oosthuizen et al. (2011) used separate pathogen and host microarrays to measure the transcriptome of *Aspergillus fumigatus* and human epithelial cells. The advantage of microarrays is, that they are cheap, processing of raw data is fast and well-established (Zhao et al., 2014). On the other hand, the recently developed RNA-Seq technology (Nagalakshmi et al., 2008) opened up the opportunity to study transcriptomes at a high level of accuracy and depth, also of non-model organisms. With the advent of dual RNA-Seq it became possible to measure transcriptomes of multiple species simultaneously without physical separation of cells. A promising research field for application are infection processes of mammalian cells by pathogens (Westermann et al., 2012).

Network inference is a systems biology approach which aims to reverse engineer underlying interaction networks based on gene expression data (Hecker et al., 2009). To account for dynamics in the change of gene expression, some tools reconstruct gene regulatory networks (GRNs) based on gene expression time series data (Gustafsson et al., 2005; Guthke et al., 2005; Gupta et al., 2011; Vlaic et al., 2012). Predicted networks suggest interactions for experimental validation, but can also put experimental findings in a bigger context (Smet and Marchal, 2010). While numerous tools are applied to predict single-species networks, e.g., (Bansal et al., 2006; Bonneau et al., 2006; Linde et al., 2010; Altwasser et al., 2012), few inter-species approaches have been published.

NetGenerator, a tool to infer small scale GRNs (Guthke et al., 2005; Toepfer et al., 2007; Weber et al., 2013), has been successfully applied to predict single-species GRNs (Linde et al., 2012; Ramachandra et al., 2014). NetGenerator infers gene-regulatory networks from gene expression time series data. The interactions and their strength are identified by a heuristic structure search and parameter optimization. The resulting model is described by ordinary differential equations and can be displayed as a directed network graph as well as simulated. In a pioneering study, the applicability of NetGenerator to predict PHI networks has been demonstrated (Tierney et al., 2012). However, this publication focused on the specific biological example while the requirements for data processing and for the algorithm to a broader class of PHI experiments are not discussed extensively.

Hereafter, we discuss a variety of aspects for dual RNA-Seq data acquisition and processing. Furthermore, we describe the application of the extended NetGenerator version to infer an inter-species GRN based on dual RNA-Seq data. Even though we focus on the novel technique RNA-Seq, most parts of the described workflow can be applied to microarray data. We evaluate the impact of multiple input stimuli on the inference accuracy with NetGenerator based on a benchmark example. The extended NetGenerator version handles missing data values, which we demonstrate with the same benchmark example. We further extended the algorithm and its application to consider variances in replicated measurement data. This is directly embedded in the inference process and indirectly through a robustness analysis. We applied this method to a real dual RNA-Seq data set of murine dendritic cells infected with *C. albicans* published by Tierney et al. (2012).

## 2. Results

### 2.1 Dual RNA-SEQ data

#### 2.1.1. Data acquisition

**Figure 1:**
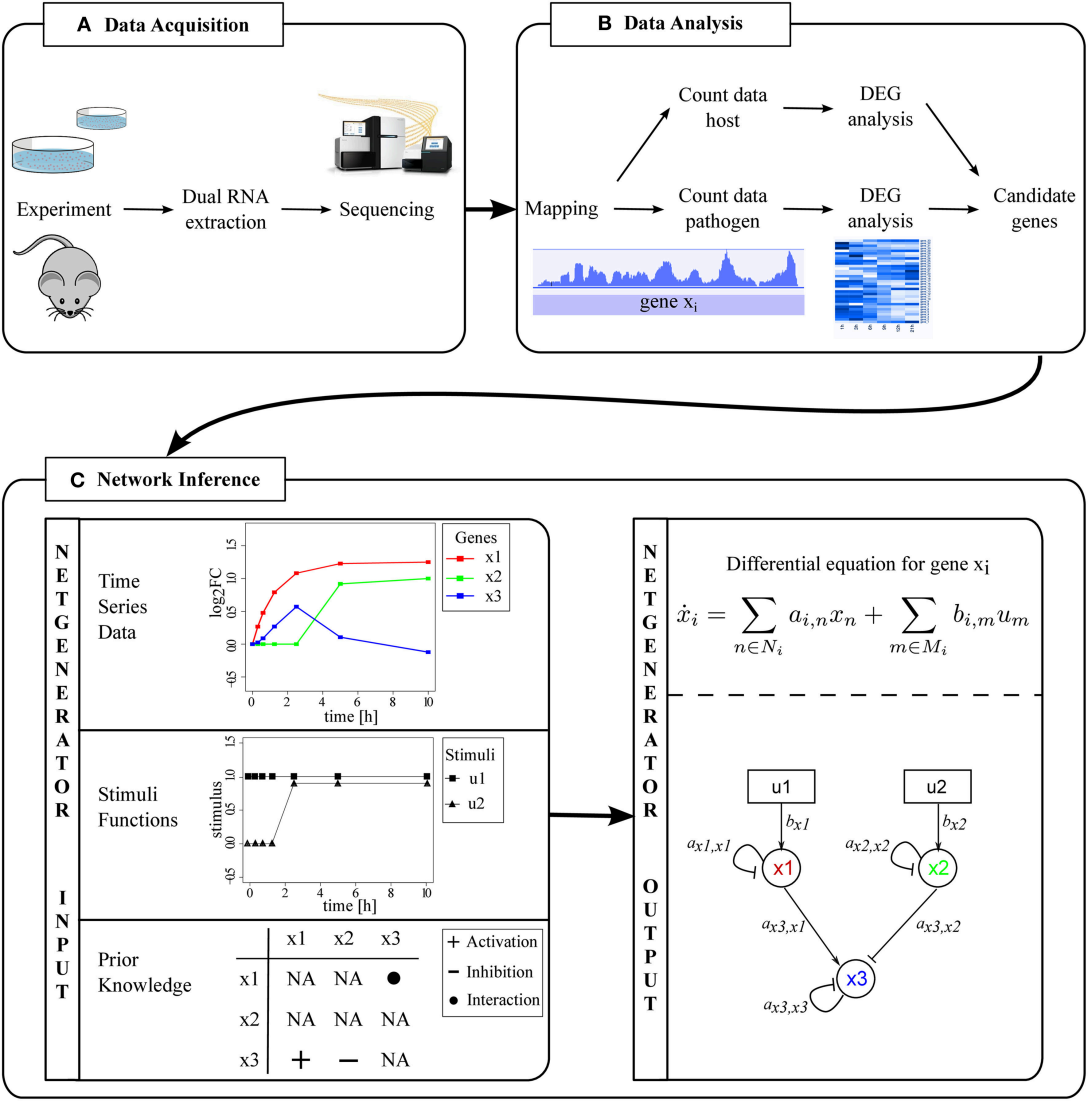
**From dual RNA-Seq data to inter-species GRNs**. **(A)** Dual RNA extraction results in one sample to be sequenced. **(B)** Data preprocessing and analysis leads to separation of host and pathogen RNA-Seq data. DEGs are identified and candidate genes selected. **(C)** Prediction of an inter-species GRN with NetGenerator.

RNA-Seq requires a certain amount of input RNA often in a microgram range, which is practically difficult to extract. Furthermore, mRNA should be enriched to avoid sequencing data being dominated by structural RNAs (Tariq et al., 2011). Additionally, the experimental setup needs to ensure that enough mRNA of both organisms can be extracted to obtain an appropriate sequencing depth (Figure 1A). Westermann et al. (2012) discuss various important limitations for dual RNA-Seq. One aspect is that different genome sizes of pathogen and host lead to different amounts of cellular RNA. It is estimated that for instance only 1.5% of the human genome encodes proteins (International Human Genome Sequencing Consortium, 2001). For that reason, we suggest to estimate an appropriate sequencing depth for both organisms based on their transcriptome sizes and recommend a genome coverage of at least 10. Tools like featureCounts return transcriptome sizes based on given annotation files as side products (Liao et al., 2014).

Furthermore, the pathogen-host cell ratio of the experimental setup, also known as multiplicity of infection (MOI), has to be considered. A high MOI results in more pathogenic RNA, but may also lead to a faster and stronger host response and less clinical relevance.

The number of reads required to achieve a good genome coverage in both species has to be estimated in advance. The number of reads needs to be calculated for the least abundant species based on the intended fold coverage, transcriptome size and read length. The total number of reads can be estimated through the ratio of the amount of extracted pathogen and host RNA.

Furthermore, sequencing parameters need to be set taking into account transcriptome sizes and how closely related studied species are. Number of reads, read length, strand-specificity and single / paired end sequencing have a great impact on the number of ambiguously mapped reads. For instance, Yazawa et al. (2013) sequenced 100-base-pair single-end reads of the grass *Sorghum bicolor* and the pathogenic fungus *Bipolaris sorghicola*. Pittman et al. (2014) sequenced 100-base-pair paired-end reads of *M. musculus* and the parasite *Toxoplasma gondii*.

Finally, data time points have to be determined. A change of the transcriptional program triggered by a stimulus is usually strong at the start of the response. Thus, in best case the organism adapts and the degree of transcriptional change decreases. The temporal onset and duration of transcriptional response of pathogen and host can be very different. To detect both responses, RNA extraction time points need to be chosen carefully. Small-scale experiments should be carried out in advance to determine good data time points.

#### 2.1.2. Dual RNA-Seq data processing

Preprocessing and analysis of sequencing data and the selection of candidate genes is an important step in advance of network inference (Figure 1B). The output of RNA-Seq are raw reads, of which low quality bases need to be trimmed [e.g., with trimmomatic (Bolger et al., 2014), btrim (Kong, 2011)]. Pathogen and host read data is separated *in silico* by aligning reads to the reference genomes (mapping). Engström et al. (2013) compare various available mapping tools and evaluate the conservative MapSplice, TopHat and STAR with comparatively low run time as favorable. From this point on, pathogen and host data are processed separately.

Tools like featureCounts (Liao et al., 2014) and htseq-Counts (Anders et al., 2015) calculate the number of reads mapped to a feature, e.g., an exon or gene, to determine gene expression levels. Subsequently, differential gene expression can be tested. Various tools [e.g., edgeR (Robinson et al., 2010), DESeq2 (Love et al., 2014)] are available for that purpose and were reviewed recently (Soneson and Delorenzi, 2013; Zhang et al., 2014). The SEQC/MAQC-III Consortium recommends to apply pipeline dependent filters for *p*-value, fold change and expression-level to decrease estimated false discovery rates. Thereby, the outputs from different differential expression analysis pipelines yield a greater agreement (SEQC/MAQC-III Consortium, 2014).

Typically, hundreds of DEGs are found, of which a subset of candidate genes has to be selected. This number can be reduced, for instance by clustering gene expression kinetics (Bezdek, 1992) and choosing one representative for each cluster. This is advantageous, because it results in a set of candidate genes representing the major expression kinetics of the system. Furthermore, gene enrichment analysis can be carried out to select functional relevant candidate genes. FungiFun2 is one of the few enrichment tools for fungi and includes 298 strains from 240 species (Priebe et al., 2015). On the other hand, many enrichment tools exist for vertebrates. The underlying algorithms can be divided into three classes of which each shows certain advantages and drawbacks. It is also recommended to apply multiple tools (Huang et al., 2009; Tipney and Hunter, 2010).

### 2.2. Modeling PHI data

We extended the heuristic network inference tool NetGenerator (see Data and Methods) and its application to predict PHI networks. NetGenerator requires logarithmic fold changes (logFCs) of gene expression time series data that can be obtained by various technologies, such as RNA-Seq or microarrays. Furthermore, the user of NetGenerator has to provide at least one input stimulus representing the external signal leading to a change in gene expression. Also, prior knowledge can be provided by the user to support the inference process (Figure 1C). It can be integrated in a compulsory (“fix”) or soft (“flexible”) way.

**Figure 2:**
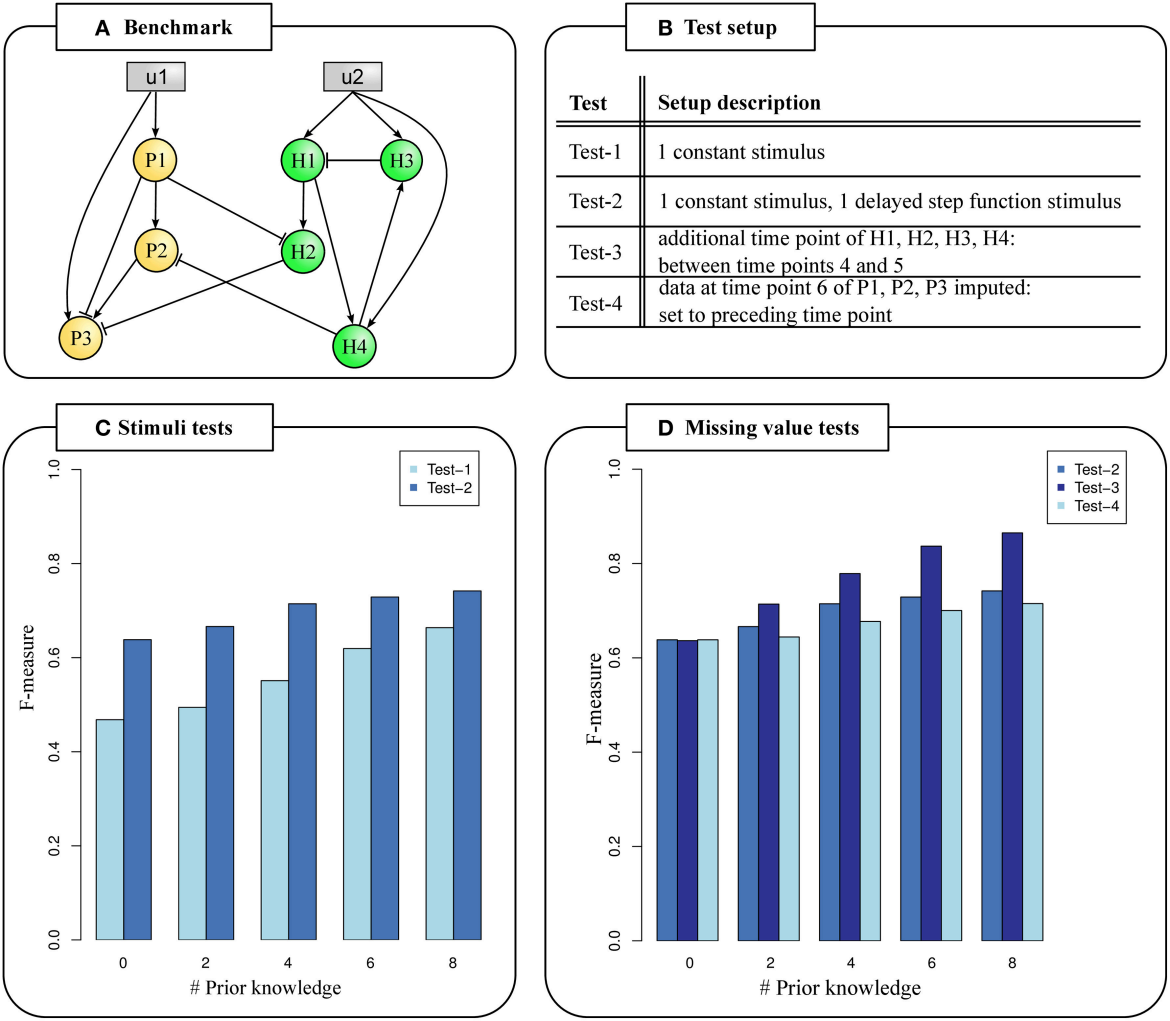
**Testing PHI data characteristics**. **(A)** Benchmark example of an inter-species GRN with 3 pathogen candidate genes (orange nodes), four host candidate genes (green nodes) and two stimuli (gray nodes). Edges represent interactions. **(B)** Test setup. **(C)** F-measures calculated from predicted network topologies and the known network topology given different stimuli functions. Two stimuli increase F-measures (Test-2). **(D)** F-measures calculated from predicted network topologies and the known network topology based on missing data values. Carefully selected time points covering both the host and pathogen response increase F-measures (Test-3).

We generated a benchmark example to evaluate the influence of different stimuli and missing data on the inference performance (see Data and Methods). The benchmark comprised six data points of seven genes and two stimuli (Figure 2A). Prior knowledge data sets of two, four, six or eight interactions were randomly generated. We applied the extended NetGenerator version to infer GRNs based on the benchmark data set and each prior knowledge data set (soft integration). For small networks as the benchmark example the number of possible solutions was already very high. On sum, 63 edges (49 gene to gene interactions and 14 stimulus to gene interactions) and 2^63^ network topologies were possible not even including the interaction sign.

#### 2.2.1. Multiple stimuli improve network inference

Multiple stimuli trigger responses in both pathogen and host during infection, such as the mutual stimulation of pathogen and host. This can be translated into at least two stimuli—the host stimulating the pathogen and *vice versa*. Weber et al. (2013) published the previous NetGenerator version V2.0 which can integrate multiple stimuli. We tested the influence of one or two stimuli on the performance of NetGenerator based on the benchmark example and each prior knowledge data set (Figure 2B).

First, only one constant stimulus (Test-1) set to a value of 1 was given. In a second test, an additional stimulus set to 0 until 30 min and set to 1 afterwards (Test-2) was given (Supplementary Table S1). We calculated mean values of F-measure (Figure 2C), sensitivity and specificity for every prior knowledge data set to determine the accuracy of predicted GRNs in comparison to the known topology (Supplementary Table S2) (see Data and Methods).

We always observed noticeable larger F-measures given two stimuli in comparison to only one given stimulus. The difference in F-measure of Test-1 and Test-2 was up to 1.36 fold (Figure 2C). The less prior knowledge was given, the larger were the differences in F-measures between Test-1 and Test-2. We found the biggest performance difference between Test-1 and Test-2 when no or only two prior knowledge interactions were given. In these cases, 15 of 21 possible true positive edges were predicted when two stimuli were given, but only 11 true positive edges given one stimulus (Supplementary Table S2). In general, we observed increasing F-measures for more given prior knowledge independent of the number of stimuli.

#### 2.2.2. Avoid missing data values

It is conceivable that time series experiments of pathogen and host were carried out independently under comparable experimental conditions. In this case, it is possible to utilize the pathogen and host data sets to predict PHI networks. Thus, data time points might differ which leads to missing values at intermediate time points or at the end of the time series. In case of dual RNA-Seq, pathogen and host are collectively processed. This may lead to a reduced amount of sample RNA of either of the species resulting in missing gene expression data. This is a problem especially for later time points when one species may dye. We extended the NetGenerator algorithm to handle missing data values at intermediate time points (see Data and Methods). We evaluated the influence of missing data on the performance based on the benchmark example, prior knowledge data sets and two given stimuli as in Test-2 (Figure 2B). Again, we calculated F-measure (Figure 2D), sensitivity and specificity (Supplementary Table S2).

We included data of one additional time point (165 min) for host genes, but additional data for pathogen genes were not given (Test-3). Thereby, we demonstrated the applicability of the extended NetGenerator version to data with missing values. We set the time point in such a way, that an additional data point covering the onset of the host response was provided and observed a noticeable increase of F-measure (Figure 2D). The difference in F-measure is greatest with 0.12 for eight given prior knowledge interactions. In this case, a mean number of 16.7 (Test-2) and 19.2 (Test-3) out of 21 possible true positive edges were predicted representing an improvement of 11.9%. This pointed out the importance of good time point selection covering both the pathogen and host response in a dual transcriptome data set.

NetGenerator requires complete data for the last time point. In case of missing measurements at the end of the time range for a subset of candidate genes, their values must be obtained in a different way and provided by the user. Here, we set the last time point to its preceding value (Test-4). We found slightly greater F-measures for Test-2 in comparison to Test-4 independent of the number of given prior knowledge. We observed a maximal difference between the F-measures between Test-2 and Test-4 (0.02) given four, six and eight prior knowledge interactions (Figure 2D).

### 2.3. Incorporation of measurement variances

Various differential expression analysis tools are available that calculate fold changes from multiple replicates. However, fold changes alone cannot reflect the degree of gene- and time point specific variances. This variance might be high especially regarding complex biological systems such as PHIs where cells from two species constantly interact and change the environment. However, biological variances can be considered in the network inference process to obtain robust predictions. For this purpose, we extended and applied NetGenerator to incorporate variances within the algorithm and in an outer robustness analysis. The extended NetGenerator algorithm was applied to one of the first published dual RNA-Seq data sets (Tierney et al., 2012) (see Data and Methods).

#### 2.3.1. Extended NetGenerator algorithm incorporates measurement variances

Variances from replicated measurements were incorporated in the objective function of NetGenerator and need to be provided by the user. We calculated variances of the dual RNA-Seq data set of Tierney et al. (2012) as described (see Data and Methods).

**Figure 3:**
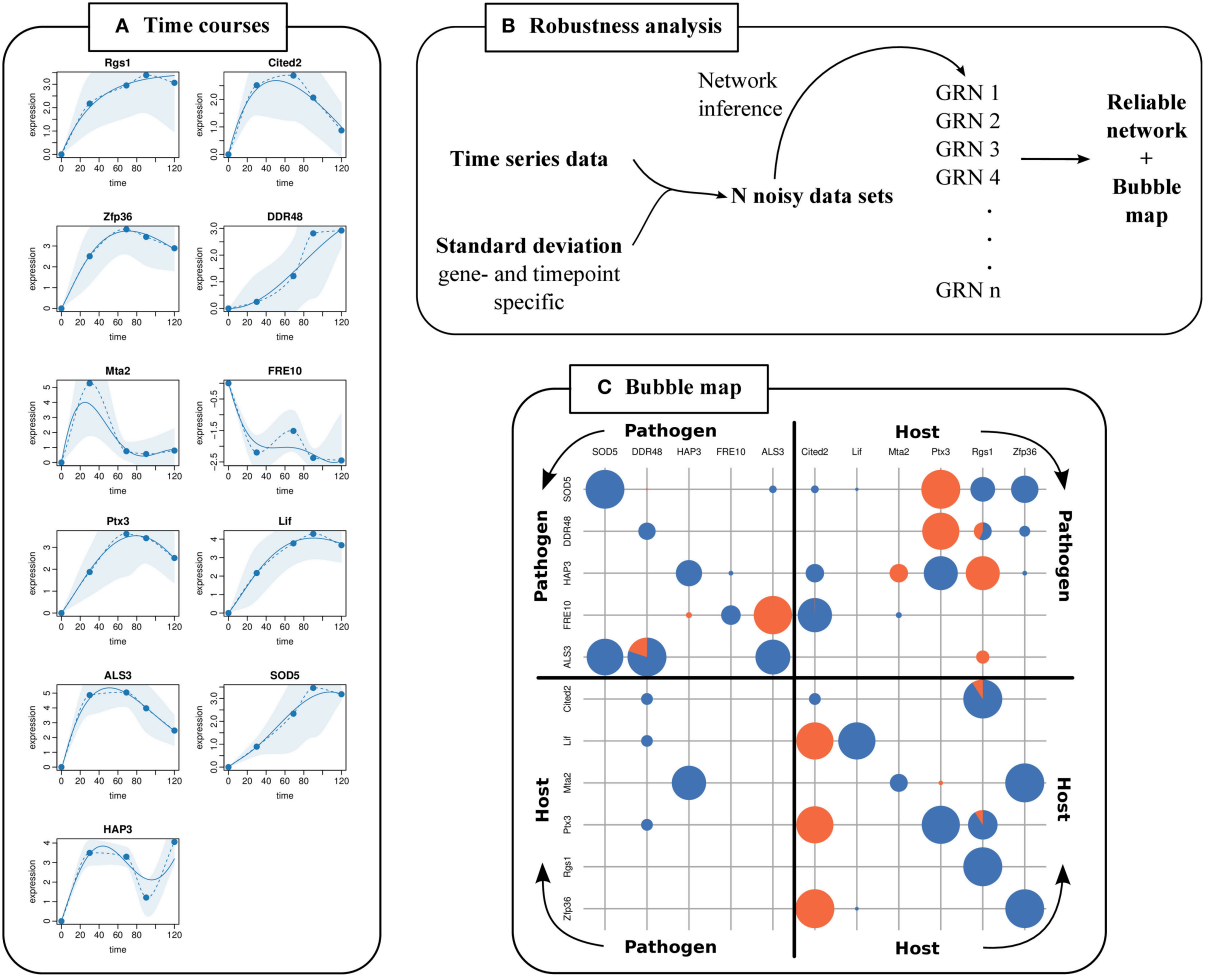
**GRN robustness analysis and visualization**. **(A)** Fitting plots for each gene are generated showing measured time points (dots), simulated time courses (solid lines), interpolated time courses (dashed lines), and standard deviations from replicated measurements (shaded areas). **(B)** Outer robustness analysis. Noise is added to time series data with variances calculated from replicates of genes and time points. This is repeated *n* times to predict *n* GRNs. **(C)** The bubble map visualizes the robustness of a predicted edge from column gene to row gene. Bubble sizes illustrate the robustness score assigned to an edge. Orange and blue pies illustrate the fraction of activating and inhibiting edges, respectively.

We predicted a GRN (Supplementary Figure S1) with the extended NetGenerator based on logFCs and prior knowledge that were used as inputs for the previous NetGenerator in Tierney et al. (2012). Calculated gene- and time point specific variances were provided as input. Measured and simulated time courses of the GRN were plotted showing the standard deviations of measurements as shaded areas (Figure 3A). We observed that simulated data reproduced the measured data very well and were mostly within the shaded areas. Furthermore, simulated time courses were closer to data points with smaller standard deviation (e.g., *Hap3* at 30 min) than to data points with higher standard deviation (e.g., *Mta2* at 30 min).

#### 2.3.2. Variance incorporation by an outer robustness analysis

Furthermore, variances were considered in an outer robustness analysis which we carried out based on the data of Tierney et al. (2012). The mean standard deviation was 1.24 with a minimum of 0.27 (*Sod5* at 120 min) and a maximum of 3.49 (*Mta2* at 30 min) (Supplementary Table S3). We scaled the standard deviations to a value of σ_max_ = 0.1 (Supplementary Table S4). We calculated Gaussian distributed logFCs for every gene and time point (mean = measured logFC, σ = scaled standard deviation of replicates) (see Data and Methods). Thus, we generated 500 noisy data sets and applied the extended NetGenerator (Figure 3B). The robustness scores of the edges in the resulting 500 GRNs were illustrated in the bubble map (Figure 3C).

Tierney et al. (2012) experimentally verified the predicted inter-species interactions of *Ptx3* inhibiting *Hap3* and *Hap3* inhibiting *Mta2*. We predicted these verified interactions again as robust with the extended NetGenerator version (Figure 3C, Supplementary Table S5). Inhibition of *Hap3* by *Ptx3* was present in 71 % of predicted GRNs with a robustness score of 0.76. Inhibition of *Mta2* by *Hap3* was present in 72 % of predicted GRNs with a robustness score of 0.78. This also demonstrated the applicability of the presented robustness test.

## 3. Discussion

In this study, we propose a workflow for dual RNA-Seq data acquisition, data processing and inter-species network inference. Furthermore, we describe how to handle a different temporal onset of transcriptional changes, missing data and how to integrate variances from replicated measurements based on the extended NetGenerator algorithm.

### 

#### 3.0.3. Delayed host response in PHI data

In a dual transcriptome data set we expect the onset of the pathogen and host transcriptional response at different time points. So far, several infection-related transcriptome studies of fungi were carried out. Transcriptome data was generated already at two to three time points within 60 min after infection (Linde et al., 2012; Ramachandra et al., 2014) suggesting an early onset of the pathogen's transcriptional response. This is further supported by a mechanism called adaptive prediction, that some pathogens have evolved. Based on cues from the current environment, pathogens predict a coming change in conditions and adapt their transcriptome in advance. An appropriate adaptation of the pathogen increases its survival chances (Brunke and Hube, 2014).

On the other hand, it takes some time until the host recognizes a pathogen. Moyes et al. (2010) showed that host epithelial cells initiate a response when a certain amount of pathogens exceeding a threshold is recognized. This is also a protective mechanism. Furthermore, the assumption of a later onset of the host transcriptional response is supported by various studies monitoring the host transcriptome from 1 h onwards (Banchereau et al., 2014; Favila et al., 2014).

However, we do not see a delayed transcriptional response of host DEGs in comparison to pathogen DEGs in the data set of Tierney et al. (2012) possibly because of the high MOI. Experimentalists keep improving their procedures to achieve realistic experimental setups, e.g., they decrease the MOI as much as possible still allowing them to extract the required amount of RNA for sequencing. Therefore, we expect to see a delayed host response in upcoming dual RNA-Seq data sets. To test the performance of the extended NetGenerator regarding different stimuli functions and missing data values, we generated a benchmark example showing a delayed onset of the host transcriptional response.

#### 3.0.4. Gene expression time series data

NetGenerator requires time series gene expression data, at least one stimulus function and optionally prior knowledge. LogFCs are passed to NetGenerator in form of a data matrix, where columns correspond to candidate genes and rows to measured time points.

PHIs are very complex systems, but available data is limited regarding the number of time points and replicates. Furthermore, transcriptome data do not provide any information about processes taking place as for instance on protein level and in the extracellular space. Therefore, it has to be considered that predicted PHIs are indirect, when they are interpreted.

#### 3.0.5. Modeling PHI stimuli

A GRN can be understood as a biological system that adapts to external, environmental stimuli yielding changes in gene expression. NetGenerator can integrate multiple stimuli and requires one function per stimulus representing it.

Many biological processes can be interpreted as external stimuli triggering responses in both pathogen and host cells during infection. In a typical experimental setup the host is incubated with the pathogen stimulating both organisms. The host recognizes PAMPs on pathogen cell surfaces by pathogen recognition receptors (PRRs). This initiates an information flow through signaling cascades (Akira et al., 2006). Nevertheless, the process of pathogen recognition resulting in a transcriptional response requires some time. Besides the molecular interaction with the host, the pathogen is also stimulated by different environmental factors, e.g., a change of temperature, pH and ion concentrations (Linde et al., 2010).

We found that multiple stimuli functions improve network inference results significantly. Therefore, we recommended to provide two or more stimuli functions for inter-species network inference. One option to model the stimulus representing the influence of the host on the pathogen is a constant function. Therewith, the stimulus is active from time point zero onwards and models an early pathogen transcriptional response. *Vice versa*, a second stimulus can represent the stimulation of the host by the pathogen. We predicted GRNs providing an additional input signal as a delayed step function (Test-2) aiming to model a later onset of the host transcriptional response. Another possible scenario would be to provide a stimulus function representing a slow increase of the influence.

More options for stimuli functions are possible when real experiments are carried out. For example, the number of differentially expressed host and pathogen genes can be determined for every time point and translated into stimuli functions. This can be done by scaling the number of DEGs to a range from zero to one. Additional measurements, e.g., cytokine release or cell contacts, can also be used as a basis for stimuli functions. Of particular interest is the growth curve of the pathogen, which we recommend to measure and integrate in the stimuli functions. Nevertheless, many biological events trigger responses, of which not all can be integrated in the network inference.

#### 3.0.6. Prior knowledge sources

Optionally, the user of NetGenerator can provide prior knowledge about interactions of candidate genes. This is strongly recommended to reduce the search space resulting from the large number of possible interactions (Hecker et al., 2009). Prior knowledge can be softly integrated by assigning a score between zero and one that reflects its reliability. A score smaller than one allows prior knowledge to be rejected if it does not fit the data.

Prior knowledge about interactions in GRNs originates from published results that were transferred to databases. PHI databases like PHISTO (Tekir et al., 2013), PHI-base (Winnenburg et al., 2006), and HPIDB (Kumar and Nanduri, 2010) have been established. Mukherjee et al. (2013) listed various web sources of interaction data.

Host specific prior knowledge can be extracted manually from literature or automatically with text mining tools. Pathway Studio is a text mining tool specific for mammals (Nikitin et al., 2003). Further gene information is provided by organism specific websites, e.g., the human gene database GeneCards^1^.

As well, organism specific websites exist for pathogens, e.g., Aspergillus Genome Database (Cerqueira et al., 2014) and Candida Genome Database (Inglis et al., 2012). To our knowledge, no fungi specific text mining tool is available. More general tools like GeneView—a semantic search engine for PubMed—can be applied (Thomas et al., 2012). Little is known about some pathogenic species. In this case, prior knowledge can be generated by searching orthologous genes in closely related and better studied organisms.

For both host and pathogen transcription factor binding motifs and binding sites can be obtained from databases, e.g., TRANSFAC (Matys et al., 2006), or predicted with bioinformatic tools as SiTaR (Fazius et al., 2011).

#### 3.0.7. Robustness analysis

We extended the NetGenerator algorithm and its application to incorporate variances from replicated measurements in the inference method and in a robustness analysis. The output provides guidance for experimental validation of predicted interactions.

Inference methods should take into account the variance of replicates, because this additional information improves the parameter estimation. Under the assumption of independent Gaussian distributed noise the minimization of the objective function (Equation 4) corresponds to a Maximum Likelihood Estimator (MLE) (see e.g., Klipp et al., 2009, p. 155). Here, we assume that the variances of each gene and time point exhibit those statistical properties sufficiently. The extended NetGenerator version incorporates available measurement variances thus providing more reliable inference results. Nevertheless, the option to predict GRNs without providing variances is still available.

In previous publications a similar robustness analysis was carried out with the same standard deviation for each gene and time point set to a fixed value (Linde et al., 2010, 2012). Biological replicates can show high variance, that is gene- and time point specific and has a great influence on the estimated fold changes as well as their significance. Both the extended objective function (Equation 4) and the robustness analysis incorporate variances. They should be determined based on the available data to account for differences between genes and time points. One possibility is the rather simple approach to calculate the total variance of a logFC from sample variances as proposed (Equation 6). Another possibility is to derive the variances from software packages that take into account the statistical nature of the measurement method (including both biological and technical variances), perform processing steps, test for significant changes and determine logFCs. For instance, the R-package DESeq2 calculates standard errors for estimated logFCs (Love et al., 2014). Since those methods adjust the variances based on a statistical foundation, the inference results can be expected to further improve.

We performed the robustness analysis for the data of Tierney et al. (2012). In the data we observed very high variances for the replicates of some genes and time points. Applying the outer robustness analysis to noisy data sets based on unscaled standard deviations led to the prediction of diverse GRNs without more frequent edges. Therefore, we scaled the set of standard deviations to a maximal value. It is preferable to decrease the variance of expression mean by generating more biological replicates (Blainey et al., 2014).

The application of the robustness analysis is beneficial in many ways. It provides a ranking of predicted interactions based on noise added to the data. This makes it easier to decide, which predicted interactions should be experimentally verified. Furthermore, NetGenerator is a heuristic algorithm, which means that not all possible solutions are tested. It is likely, that not the best solution is returned, but a good one. The robustness analysis generates many good solutions resulting in a consensus network. It also accounts for possible mutually contradictory predictions.

## 4. Data and methods

### 4.1. Application of extended NetGenerator to PHI data

Network inference was carried out by the NetGenerator algorithm (see Guthke et al., 2005; Toepfer et al., 2007; Weber et al., 2013 for details). For this study, the previous NetGenerator V2.0 was extended (recent version of the R package: 2.3-0) to account for measurement variances and missing values.

#### 4.1.1. Basic algorithm

The NetGenerator heuristics infers GRNs from time series gene expression data of multiple experiments and multiple stimulation. Expression data (logFCs), stimuli functions and prior knowledge (optionally) have to be provided by the user. Stimuli are factors that (directly or indirectly) cause changes in gene expression. It is assumed, that stimuli are not influenced by genes or their products, at least in the experimental setup. Nevertheless, stimuli values may evolve over time.

The inferred network model is described by a system of first order linear differential equations of the form

(1) $$\underline{\dot{x}}=\underline{\underline{A}}\underline{x}+\underline{\underline{B}}\underline{u}.$$

The change of gene expression *ẋ* is influenced by other genes and (external) stimuli *u*. While interactions between genes are described by the system matrix $\underline{\underline{A}}$ : *N* × *N*, the influence of stimuli is represented by the input matrix $\underline{\underline{B}}$ : *N* × *M*, where *N* is the number of genes and *M* is the number of inputs. The inference procedure determines the elements of these matrices, i.e., the parameters θ of the model, by an iterative heuristics including structure and parameter optimization. In each iteration step, the algorithm includes a submodel which matches the available time series data best. The parameters of the *i*th submodel are determined by minimizing an objective function

(2) $$J_{i}=J_{i,\text{output}}+J_{i,\text{priorknowledge}}$$

The second term evaluates the integration of prior knowledge, see (Weber et al., 2013) for details. In previous NetGenerator versions the first term

(3) $$J_{i,\text{output}}=\sum_{e=1}^{E}\sum_{k=1}^{T_{e,i}}\left[w(t_{k})\times {\left(x_{e,i}(t_{k})-{\hat{x}}_{e,i}(t_{k},{\underline{\theta }}_{i})\right)}^{2}\right]$$

described the error between measured data *x* and simulated data $\hat{x}$. The double sum was calculated for all experiments *E* and all time points *T_e,i_*. Since the data contain both real and interpolated artificial values, this was accounted for by weighting factors *w*(*t_k_*).

#### 4.1.2. Extension to account for missing values

NetGenerator was extended to account for missing data values. Now, NetGenerator accepts missing values at intermediate time points provided by the user as “NA.” Internally, the time vector of the respective output is adjusted and interpolation is carried out based on existing measurement data. During inference, both simulation and objective function (Equation 4) can process that information of missing and replaced values.

#### 4.1.3. Extension to incorporate variances

The objective function *J_i,output_* (Equation 3) was extended by additional weighting factors, which are the reciprocal variances 1/σ^2^ of the replicated data:

(4) $$J_{i,\text{output}}=\sum_{e=1}^{E}\sum_{k=1}^{T_{e,i}}\left[\frac{w(t_{k})}{\sigma_{e,i}^{2}(t_{k})}\times {\left(x_{e,i}(t_{k})-{\hat{x}}_{e,i}(t_{k},{\underline{\theta }}_{i})\right)}^{2}\right]$$

Therefore, the variances σ^2^ of the logFCs became additional input arguments to NetGenerator. Larger variances decrease the objective function value which effectively allows for a larger error between associated measured and simulated values in comparison to measurements of smaller variance.

#### 4.1.4. Incorporation of variances in an outer robustness analysis

Moreover, variances are considered in an outer robustness analysis by predicting GRNs based on disturbed logFCs. To simulate the measurement process, we sampled three replicates of Gaussian distributed logFCs (mean = measured logFC, σ = standard deviation of replicates) and determined their mean. This resulted in a noisy logFC for each candidate gene and time point used as input for extended NetGenerator. We repeated this process 500 times.

For better visualization of the robustness analysis results we introduced the bubble map (Figure 3C) showing predicted interactions between candidate genes. It does not only consider the occurrence frequency of each edge, but also the sign and the respective objective function values *J* = ∑ *J_i_* that is the sum over the values of each time series (Equation 2). The robustness score *S_i,j_* evaluating the interaction of gene *j* and gene *i* is calculated as

(5) $$S_{i,j}=\sum_{k}\left\{\frac{1}{J_{i,j,k}}|a_{i,j,k}\neq 0\right\}$$

with *J_i,j,k_* being the objective function value of the *k^th^* predicted GRN and *a_i,j,k_* being the corresponding element of the interaction matrix $\underline{\underline{A}}$. A robustness score *S_i,j_* of gene *j* interacting with gene *i* is illustrated by the bubble size of column *j* and row *i* (Figure 3C).

A big circle represents a frequently predicted interaction. Small or no circles represent rarely or no predicted interactions. Pie charts show the ratio of inferred activating (orange) and inhibiting (blue) interactions. Note, that the diagonal represents autoregulations. Exact robustness scores depending on how frequently an edge was predicted and corresponding objective function values of the predicted GRN are available as additional output (Supplementary Table S5).

#### 4.1.5. Calculation of variances from replicates

Both the extended version of the objective function and the robustness analysis require variances derived from data. The gene- and time point specific variance σ^2^_*tc*_ of each logFC was calculated as the variance of the difference μ_*t*_ − μ_*c*_ between means of treatment (t) and control (c) samples (error propagation):

(6) $$\sigma_{tc}^{2}=\sigma_{c}^{2}+\sigma_{t}^{2}$$

The respective standard deviations σ_*i,j*_ of all genes and time points can be obtained by taking the square root of the variances. Given only few replicates, standard deviations can be high leading to the prediction of diverse GRNs. In that case, the standard deviations need to be scaled to a maximal value σ_max_:

(7) $$\sigma_{i,j,\text{scaled}}=\frac{\sigma_{i,j}\times \sigma_{\text{max}}}{max(\underline{\underline{\sigma }})}$$

### 4.2. Data sets and evaluation criteria

#### 4.2.1. Benchmark model

We constructed a benchmark system composed of differential equations representing the logFC time series data of three pathogen genes, four host genes and two stimuli. The network topology included 21 directed, signed edges representing interactions. Common biological motifs like feed forward loops and feedback loops are integrated, too. Based on this topology we set up a system of differential equations and simulated this model with the R-package deSolve (Soetaert et al., 2010). We set the time point 0 min to zero and extracted data values of every differential equation at six time points on a logarithmic scale (15, 30, 60, 120, 250, 500 min). We added Gaussian distributed noise (*mean* = 0, σ = 0.01) to generate the benchmark data set.

As mentioned before, an additional input to guide network inference is prior knowledge. We generated a prior knowledge data set for the benchmark data by randomly sampling two interactions of the known network topology and repeated this 50 times. 50% of sampled prior knowledge is signed (activation or inhibition) and 50% is unspecific. Likewise, we generated prior knowledge data sets of four, six and eight interactions.

To evaluate predicted GRNs we computed statistical measures that compare the known topology to the predicted topology. Sensitivity (SE), specificity (SP), precision (PR) and F-measure (FM) are calculated as:

(8) $$\begin{array}{l} \;SE=TP/(TP+FN+FP_{\text{s}})\\ \;SP=TN/(TN+FP_{\text{n}})\\ \;PR=TP/(TP+FP_{\text{n}}+FP_{\text{s}})\\ FM=(2\times PR\times SE)/(PR+SE) \end{array}$$

taking the number of true positives (TP), false positives not part of the known topology (FP_n_), false positives with wrong sign (FP_s_), true negatives (TN) and false negatives (FN) into account (Weber et al., 2013). All of these statistical measures range from zero to one with one evaluating a predicted network as identical to the known topology.

#### 4.2.2. Real dual RNA-Seq data

We utilized one of the first dual RNA-Seq data sets published by Tierney et al. (2012) as a second data set for evaluation. Murine dendritic cells were infected with *C. albicans* (*MOI* = 5). Three biological replicates were generated at 0, 30, 60, 90, 120 min after infection. Differential expression analysis was carried out with DESeq (Tierney et al., 2012). Six murine DEGs and five fungal DEGs were selected as candidate genes to predict an inter-species GRN with NetGenerator V1.0 (Toepfer et al., 2007). 19 prior knowledge edges were provided and softly integrated. We reproduced the result with NetGenerator V2.0 (Weber et al., 2013) based on the logFCs, stimulus function and prior knowledge of Tierney et al. (2012). Furthermore, we applied DESeq to determine logFCs and normalized count values to calculate gene- and time point specific variances.

The predicted interactions of *Ptx3* inhibiting *Hap3* and *Hap3* inhibiting *Mta2* were experimentally verified by Tierney et al. (2012). Therefore, these two interactions should be again predicted by the extended NetGenerator and were thus used for evaluation.

#### 4.2.3. Availability

The extended NetGenerator 2.3.-0 tool is available at http://www.biocontrol-jena.com/NetGenerator/NetGenerator_2.3-0.tar.gz.

## Author contributions

Conception and design of the investigation and work: all. Analyzing the properties of PHIs: Sylvie Schulze, Jörg Linde, and Reinhard Guthke. Implementation of NetGenerator and contribution to mathematical background: Sebastian G. Henkel and Dominik Driesch. Data processing, application of computational algorithm and evaluation of results: Sylvie Schulze, Sebastian G. Henkel, and Jörg Linde. Drafting the manuscript: Sylvie Schulze and Sebastian G. Henkel. Revising it critically for important intellectual content and final approval of the version to be published: all. Agreement to be accountable for all aspects of the work in ensuring that questions related to the accuracy or integrity of any part of the work are appropriately investigated and resolved: all.

## Funding

Sylvie Schulze and Jörg Linde are supported by the Deutsche Forschungsgemeinschaft (DFG) CRC/Transregio 124 “Pathogenic fungi and their human host: Networks of interaction,” subproject B3 (Sylvie Schulze) and subproject INF (Jörg Linde). Sebastian G. Henkel and Dominik Driesch are supported within the Virtual Liver Network funded by the German Federal Ministry of Education and Research (BMBF, Fkz. 0315760).

### Conflict of interest statement

The authors declare that the research was conducted in the absence of any commercial or financial relationships that could be construed as a potential conflict of interest.

## Footnotes

^1^www.genecards.org
