# Supplementary material for: Computational prediction of molecular pathogen-host interactions based on dual transcriptome data
Source: Front Microbiol. 2015 Feb 6;6:65. doi: 10.3389/fmicb.2015.00065 (PMC4319478; doi:10.3389/fmicb.2015.00065)
Supplement: Supplementary file 1 [file DataSheet1.PDF]

Supplementary Material: Computational prediction of molecular pathogen-host interactions based on dual transcriptome data

Sylvie Schulze [1], Sebastian G. Henkel [2], Dominik Driesch [2], Reinhard Guthke [1] and Jörg Linde [1,*]

[1] *Research Group Systems Biology and Bioinformatics, Leibniz-Institute for Natural Product Research and Infection Biology - Hans-Knoell-Institute, Jena, Germany*
[2] *BioControl Jena GmbH, Jena, Germany*

Correspondence*:
Dr. Jörg Linde
[1]Research Group Systems Biology and Bioinformatics, Leibniz-Institute for Natural Product Research and Infection Biology - Hans-Knoell-Institute,
Beutenbergstr. 11a, 07745 Jena, Germany, joerg.linde@hki-jena.de

1 SUPPLEMENTARY TABLES AND FIGURES

Supplementary Table S1. Stimuli functions utilized to test their influence on the inference performance.

	Stimulus 1	Stimulus 2
0 min	1	0
15 min	1	0
30 min	1	0
60 min	1	1
120 min	1	1
250 min	1	1
500 min	1	1

Supplementary Table S2. For each test and prior knowledge data set the predicted network topology is compared to the known network topology. Average numbers of true positives (TP), true negatives (TN), false positives (FP), false negatives (FN), sensitivity, specificity and F-measure are calculated.

	TP	TN	FP	FN	Sensitivity	Specificity	F-measure
Test-1_noPK	11	23	11	8	0.5	0.676	0.468
Test-1_pk2	11.489	23.844	10.156	7.667	0.522	0.701	0.494
Test-1_pk4	12.857	24.543	9.457	6.829	0.584	0.722	0.551
Test-1_pk6	14.439	25.561	8.439	5.829	0.656	0.752	0.619
Test-1_pk8	15.617	26.149	7.851	4.809	0.71	0.769	0.664
Test-2_noPK	15	31	10	7	0.682	0.756	0.638
Test-2_pk2	15.447	32.191	8.809	6.426	0.702	0.785	0.666
Test-2_pk4	16.422	33.6	7.4	5.4	0.746	0.82	0.715
Test-2_pk6	16.581	34.442	6.558	5.047	0.754	0.84	0.729
Test-2_pk8	16.717	35.261	5.739	4.674	0.76	0.86	0.742
Test-3_noPK	14	33	8	8	0.636	0.805	0.636
Test-3_pk2	15.617	35.128	5.872	6.106	0.71	0.857	0.714
Test-3_pk4	17.174	36.326	4.674	4.565	0.781	0.886	0.779
Test-3_pk6	18.386	37.705	3.295	3.341	0.836	0.92	0.837
Test-3_pk8	19.19	38.238	2.762	2.381	0.872	0.933	0.865
Test-4_noPK	15	31	10	7	0.682	0.756	0.638
Test-4_pk2	14.88	31.92	9.08	6.86	0.676	0.779	0.644
Test-4_pk4	15.478	33.326	7.674	5.913	0.704	0.813	0.677
Test-4_pk6	15.786	34.5	6.5	5.357	0.718	0.841	0.7
Test-4_pk8	16	35.093	5.907	5.14	0.727	0.856	0.715

Supplementary Table S3. Standard deviations calculated from replicated measurements for every candidate gene and time point.

	SOD5	DDR48	HAP3	FRE10	ALS3	Cited2	Lif	Mta2	Ptx3	Rgs1	Zfp36
0 min	0	0	0	0	0	0	0	0	0	0	0
30 min	0.401	1.051	0.652	0.548	1.33	1.087	1.466	3.487	1.099	1.229	1.359
60 min	1.636	1.391	0.514	0.66	0.977	1.654	1.184	0.629	1.947	1.383	1.201
90 min	2.161	2.443	0.941	0.302	1.636	1.306	1.373	0.977	1.155	1.626	1.386
120 min	0.274	0.637	1.217	1.506	1.052	0.986	0.935	1.47	1.141	2.104	1.109

Supplementary Table S4. Standard deviations calculated from replicated measurements for every candidate gene and time point. Values are scaled to a maximal value of 0.1.

	SOD5	DDR48	HAP3	FRE10	ALS3	Cited2	Lif	Mta2	Ptx3	Rgs1	Zfp36
0 min	0	0	0	0	0	0	0	0	0	0	0
30 min	0.011	0.03	0.019	0.016	0.038	0.031	0.042	0.1	0.032	0.035	0.039
60 min	0.047	0.04	0.015	0.019	0.028	0.047	0.034	0.018	0.056	0.04	0.034
90 min	0.062	0.07	0.027	0.009	0.047	0.037	0.039	0.028	0.033	0.047	0.04
120 min	0.008	0.018	0.035	0.043	0.03	0.028	0.027	0.042	0.033	0.06	0.032

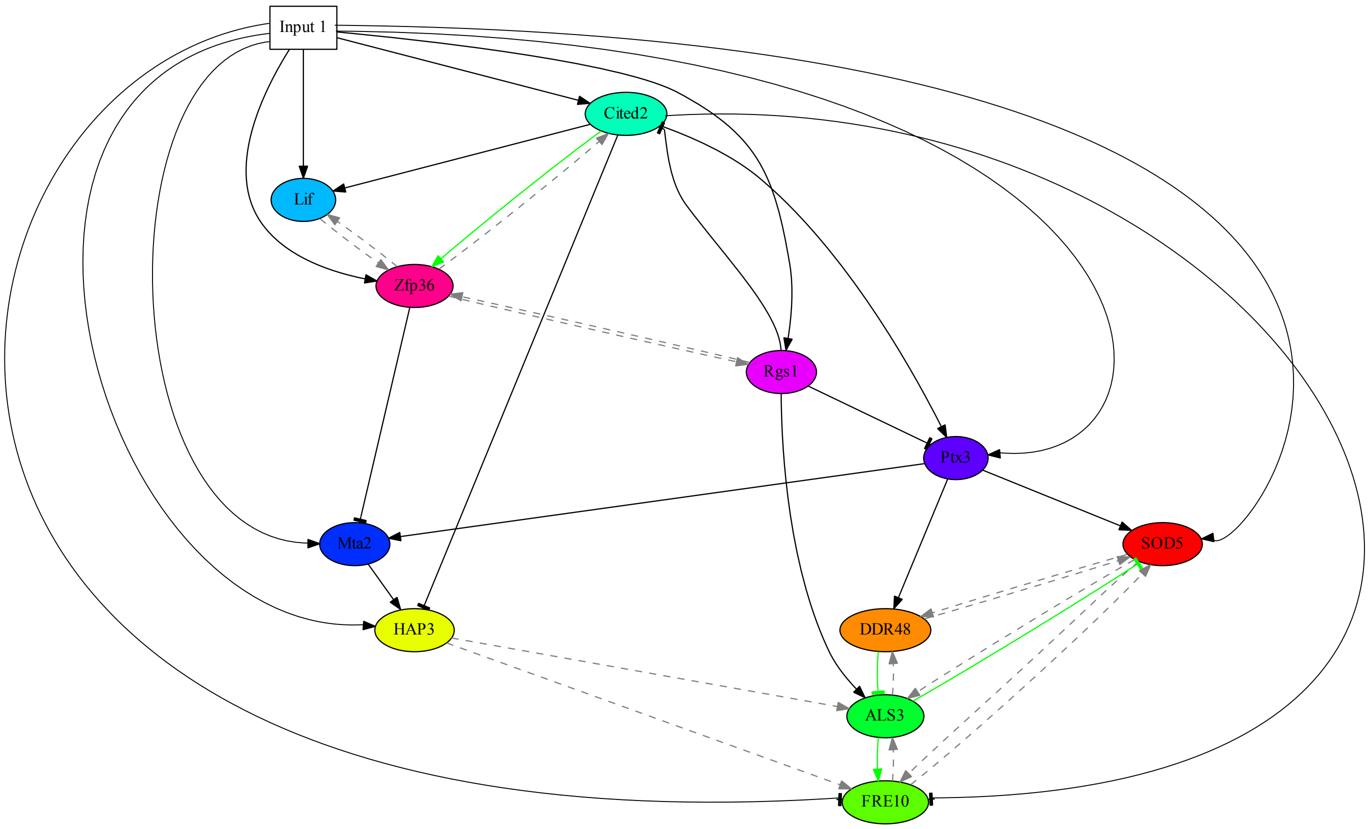

Supplementary Figure S1. Predicted GRN given variances calculated from replicated data as well as logFCs and a constant stimulus function retrieved from **Tierney et al.** (2012). The network contains 11 genes (nodes) and one simulated input (square). Shown are newly predicted interactions (black), predicted interactions based on prior knowledge (green) and given prior knowledge interactions not predicted (grey dashed).

Supplementary Table S5. Results of the robustness analysis. Detailed information about the predicted influence of the regulator gene on the target gene is listed: the relative frequency of occurence of activating (Activation.rFoC) and inhibiting (Inhibition.rFoC) edges, the weighted proportion of activating (Activation.wProportion) and inhibiting (Inhibition.wProportion) edges shown in the bubble map and the robustness score of an edge corresponding to the bubble size.

Regulator	Target	Activation.rFoC	Inhibition.rFoC	Activation.wProportion	Inhibition.wProportion	Score
ALS3	ALS3	0	0.846	0	1	0.8016660541
ALS3	FRE10	0.968	0.002	0.9975168221	0.0024831779	0.9686669107
ALS3	SOD5	0	0.034	0	1	0.0369002137
Cited2	Cited2	0	0.106	0	1	0.0911531921
Cited2	FRE10	0.002	0.75	0.0031096428	0.9968903572	0.7735204533
Cited2	HAP3	0	0.276	0	1	0.2233929402
Cited2	Lif	0.896	0	1	0	0.9107290992
Cited2	Ptx3	0.894	0	1	0	0.9088468079
Cited2	SOD5	0	0.05	0	1	0.0390856072
Cited2	Zfp36	1	0	1	0	1
DDR48	ALS3	0.154	0.844	0.1987776594	0.8012223406	0.9977677897
DDR48	Cited2	0	0.106	0	1	0.0911531921
DDR48	DDR48	0	0.182	0	1	0.1963103853
DDR48	Lif	0	0.104	0	1	0.0892709008
DDR48	Ptx3	0	0.106	0	1	0.0911531921
DDR48	SOD5	0.002	0	1	0	0.0027245758
FRE10	FRE10	0	0.214	0	1	0.2509555171
FRE10	HAP3	0	0.012	0	1	0.0145194568
HAP3	FRE10	0.02	0	1	0	0.022583482
HAP3	HAP3	0	0.414	0	1	0.4517676212
HAP3	Mta2	0	0.724	0	1	0.7766070598
Lif	Lif	0	0.896	0	1	0.9107290992
Lif	SOD5	0	0.008	0	1	0.0072168434
Lif	Zfp36	0	0.008	0	1	0.0089709834
Mta2	FRE10	0	0.02	0	1	0.022583482
Mta2	HAP3	0.276	0	1	0	0.2233929402
Mta2	Mta2	0	0.26	0	1	0.2105407083
Ptx3	DDR48	0.894	0	1	0	0.9088468079
Ptx3	HAP3	0	0.712	0	1	0.762087603
Ptx3	Mta2	0.016	0	1	0	0.0128522319
Ptx3	Ptx3	0	0.948	0	1	0.9596531763
Ptx3	SOD5	1	0	1	0	1
Rgs1	ALS3	0.114	0	1	0	0.1146830967

Supplementary Table S5. Results of the robustness analysis. Detailed information about the predicted influence of the regulator gene on the target gene is listed: the relative frequency of occurence of activating (Activation.rFoC) and inhibiting (Inhibition.rFoC) edges, the weighted proportion of activating (Activation.wProportion) and inhibiting (Inhibition.wProportion) edges shown in the bubble map and the robustness score of an edge corresponding to the bubble size.

Regulator	Target	Activation.rFoC	Inhibition.rFoC	Activation.wProportion	Inhibition.wProportion	Score
Rgs1	Cited2	0.106	0.894	0.0911531921	0.9088468079	1
Rgs1	DDR48	0.106	0.09	0.4370482959	0.5629517041	0.2085654902
Rgs1	HAP3	0.712	0	1	0	0.762087603
Rgs1	Ptx3	0.054	0.476	0.0885035158	0.9114964842	0.5740604523
Rgs1	Rgs1	0	1	0	1	1
Rgs1	SOD5	0	0.436	0	1	0.4017196624
SOD5	ALS3	0	0.886	0	1	0.8853169033
SOD5	SOD5	0	0.992	0	1	0.9927831566
Zfp36	DDR48	0	0.062	0	1	0.0780379302
Zfp36	HAP3	0	0.012	0	1	0.0145194568
Zfp36	Mta2	0	1	0	1	1
Zfp36	SOD5	0	0.454	0	1	0.4915210036
Zfp36	Zfp36	0	0.992	0	1	0.9910290166

REFERENCES

Tierney, L., Linde, J., Müller, S., Brunke, S., Molina, J. C., Hube, B., et al. (2012), An interspecies regulatory network inferred from simultaneous RNA-Seq of *Candida albicans* invading innate immune cells, *Frontiers in Microbiology*, 3, doi:10.3389/fmicb.2012.00085
